# Virtual Screening of Potential RoxS Inhibitors and Evaluation of Their Antimicrobial Activity in Combination with Antibiotics against Clinically Resistant Bacteria

**DOI:** 10.3390/antibiotics12091422

**Published:** 2023-09-08

**Authors:** Ya-Yan Huang, Jia-Hao Li, Ting-Ting Liang, Ze-An Zhao, Jun Xu, Wen-Ying Chen

**Affiliations:** 1 Department of Pharmacy, The Third Affiliated Hospital, Southern Medical University, Guangzhou 510630, China; 2 College of Pharmacy, Jinan University, Guangzhou 510632, China; 3 Guangdong Provincial Key Laboratory of Drug Screening, School of Pharmaceutical Sciences, Southern Medical University, Guangzhou 510515, China

**Keywords:** RoxS, homology modeling, molecular docking, molecular dynamics simulations, checkerboard assay, *Pseudomonas aeruginosa*

## Abstract

*Pseudomonas aeruginosa* with difficult-to-treat resistance has been designated as an urgent or serious threat by the CDC in the United States; therefore, novel antibacterial drugs and combination strategies are urgently needed. The sensor kinase RoxS is necessary for the aerobic growth of *Pseudomonas aeruginosa*. This study aimed to screen candidate RoxS inhibitors and evaluate their efficacy in treating multi-drug-resistant and extensively drug-resistant *Pseudomonas aeruginosa* in combination with meropenem and amikacin to identify promising combination strategies. RoxS protein structures were constructed using homology modeling and potential RoxS inhibitors, including Ezetimibe, Deferasirox, and Posaconazole, were screened from the FDA-approved ZINC drug database using molecular docking and molecular dynamics simulations. MIC and checkerboard assays were used to determine the in vitro antimicrobial efficacy of the three drugs in combination with antibiotics. The results of in vitro experiments showed an additive effect of 100 μg/mL Deferasirox or 16 μg/mL Posaconazole in combination with meropenem and a synergistic effect of 1.5 μg/mL Deferasirox and amikacin. In summary, these three drugs are potential inhibitors of RoxS, and their combination with meropenem or amikacin is expected to reverse the resistance of *P. aeruginosa*, providing new combination strategies for the treatment of clinically difficult-to-treat *Pseudomonas aeruginosa.*

## 1. Introduction

*Pseudomonas aeruginosa (P. aeruginosa)* is a rod-shaped, aerobic, Gram-negative bacterium that is an important causative agent of clinically acquired infections [1]. The incidence of life-threatening *P. aeruginosa* infections in critically ill and immunocompromised patients is increasing [2,3,4,5]. Antibiotics are the cornerstone of modern medicine, however, they also exert survival pressure on bacteria. In response to the external environment, bacteria undergo accelerated evolution leading to drug resistance [6,7]. Multidrug-resistant (MDR) and extensively drug-resistant (XDR) strains have spread worldwide and become a significant threat to public health, even in the context of combination therapy [8]. A prospective European study showed that among *P. aeruginosa* isolates from intensive care units, 32.9% were MDR and 24.9% were XDR [9]. Carbapenems and aminoglycosides are commonly used to treat severe *P. aeruginosa* infections, however, the resistance of *P. aeruginosa* to these drugs increases every year [10,11]. Therefore, the treatment of MDR and XDR *P. aeruginosa* is a global challenge [12]. There is an urgent need to search for novel antibacterial synergists by combining them with antibiotics to overcome antibiotic resistance in *P. aeruginosa*; therefore, it is necessary to develop novel antimicrobial drugs and therapeutic strategies.

A class of regulatory proteins in *P. aeruginosa*, known as the two-component system (TCS), consists of paired sensors and response regulators [13]. Several activities of *P. aeruginosa* are regulated by this mechanism, including motility, metabolic processes, and biofilm formation [14]. Therefore, TCS has emerged as a target for the development of novel antibacterial drugs [15]. *P. aeruginosa* has at least five terminal oxidases involved in aerobic respiration. *P. aeruginosa* adapts to different environmental conditions by regulating the expression of multiple oxidases and anaerobic energy metabolism [16]. Jo J et al. demonstrated that the cbb3-type cytochrome oxidase subunit supports *P. aeruginosa* biofilm growth and virulence [17]; however, an imbalance in the regulatory mechanisms may inhibit bacterial growth. Osamura T et al. reported that cytochrome c oxidase (aa3 (Cox)) is expressed at low levels during the normal growth of *P. aeruginosa*, and its overexpression significantly inhibits the aerobic growth of the bacteria [18]. RoxSR is a TCS in *P. aeruginosa* and a regulator of terminal oxidases that represses the expression of Aa3 oxidase and activates the expression of the other four terminal oxidases. Abnormal expression of RoxSR may interfere with the aerobic respiration of bacteria and reduce their adaptability to unfavorable environments, thereby inhibiting bacterial growth. Azide compounds have an inhibitory effect on bacteria; Comolli JC et al. observed that *P. aeruginosa* strains lacking RoxS were less resistant to azide than the wild type [19]. In addition, Hurley BP et al. showed that the RoxSR mutant of *P. aeruginosa* was unable to grow under 500 uM NaN3 conditions, unlike wild-type PAO1 [20]. Therefore, RoxSR may be a promising target for bacterial inhibition.

Since 2008, our group has been dedicated to reversing drug resistance in *P. aeruginosa* [21,22,23]. Jun L et al. found that interfering with bacterial iron metabolism affected bacterial drug resistance, and the metabolism was closely related to the physiological activities of bacteria [24]. RoxSR, a two-component system that affects bacterial metabolism, caught our attention. Several studies have investigated the mechanism by which RoxSR regulates aerobic respiration; however, its inhibitors have not yet been reported. Sensor kinase proteins are commonly used as targets in the development of antibacterial agents for TCS to control the biological functions of the entire TCS [25,26]. RoxS is a sensor kinase of RoxSR; therefore, it was selected as the target of this study. The time required to develop novel, effective, and safe therapies is a major barrier to the discovery of antibiotics. Repurposing FDA-approved drugs to improve the efficacy of existing antimicrobial drugs is a favorable option for expanding the existing antimicrobial drug pool. Therefore, virtual screening was used to identify potential RoxS inhibitors among FDA-approved drugs and to explore the optimal combination treatment strategy for inhibitor candidates with both meropenem (MEM) and amikacin (AMK).

## 2. Results

### 2.1. Homology Modeling

**Figure 1:**
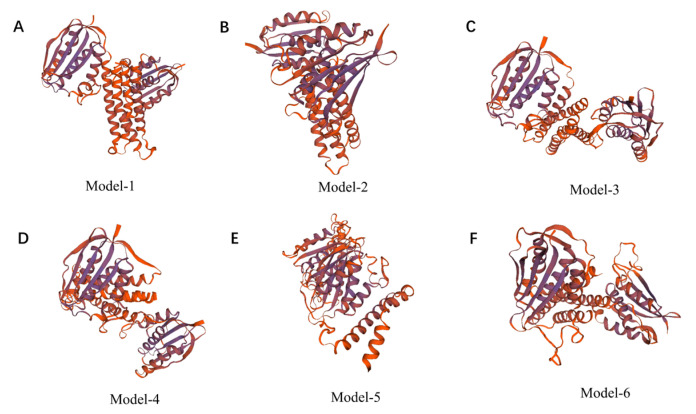
Structures of RoxS protein predicted using Swiss-Model (**A**–**F**).

A total of 50 templates were searched using the Swiss-Model, and six models were built (Figure 1). The sequence identity value represents the sequence homology, and it is required to be at least 30%. In this study, the sequence identity values for the six models were all <30% (see Table A1 of Appendix A); therefore, the protein models constructed using the Swiss-Model were of poor quality. 

**Figure 2:**
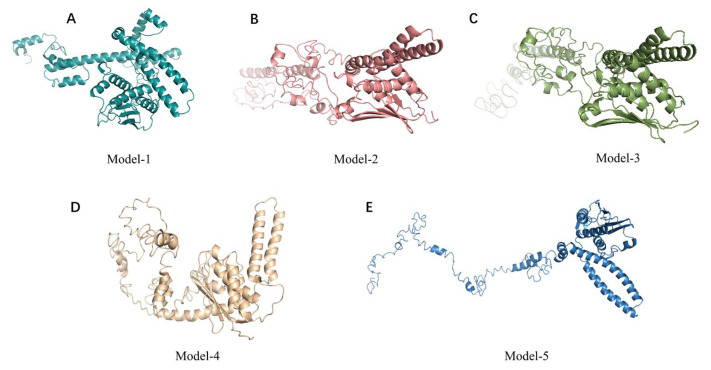
Structures of RoxS protein predicted using I-TASSER (**A**–**E**).

**Figure 3:**
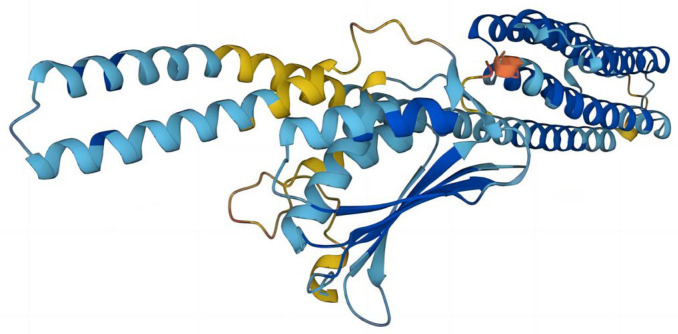
Structure of RoxS protein predicted using Alphafold. Model Confidence: Dark blue: Very high (pLDDT > 90), light blue: Confident (90 > pLDDT > 70), yellow: Low (70 > pLDDT > 50); Orange: Very low (pLDDT < 50). AlphaFold produces a per-residue confidence score (pLDDT) between 0 and 100. Some regions with low pLDDT may be unstructured in isolation.

The top five prediction models were provided by I-TASSER (Figure 2). These models are ranked by I-TASSER’s proprietary C-Score. The C-score is typically in the range of [−5.2], with higher values indicating a higher level of confidence in the model and vice versa. The C-scores of the five models in order are −1.85, −3.00, −3.35, −3.93, and −4.07, respectively. In addition to the C-score, the top-ranked model gives an Estimated TM-score = 0.49 ± 0.15, which lies between 0.17 and 0.5. TM scores above 0.5 indicate that the model has the correct topology, and TM scores below 0.17 indicate that the model is random and unreliable. The prediction models provided by I-TASSER had a low level of confidence. A protein structure of RoxS was determined using Alphafold with the biological source *P. aeruginosa* (Figure 3). The confidence level pLDDT of this model was 82.57. Further quality assessment of the protein structure models predicted by I-TASSER and Alphafold is needed.

**Figure 4:**
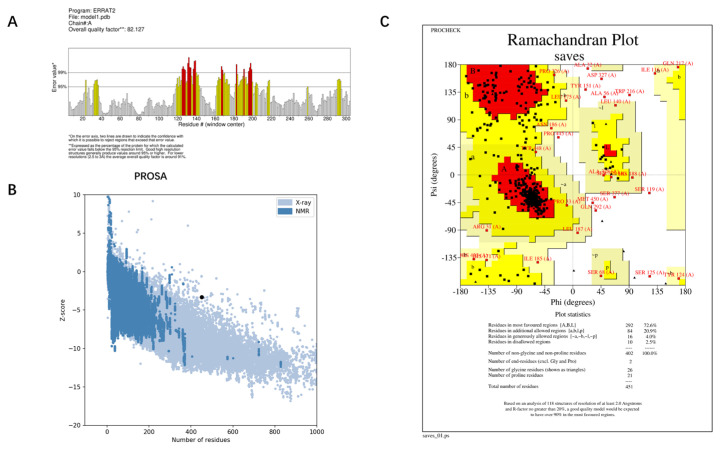
Quality evaluation results of Model 1 provided by I-TASSER: (**A**) Overall quality factor of ERRAT; (**B**) PROSA-web Z-scores of all protein chains; (**C**) PROCHECK analysis of the Ramachandran plot. The Overall quality factor of ERRAT > 95% means the structure has good resolution. The distribution of >90% of the residues in the most favorable region of the Ramachandran plot indicates that the protein structure is reasonable. The energy of the target protein structure is considered reasonable if the Z-score values of the target protein are distributed within the region of the graph represented by the Z-score values of these known proteins in PROSA.

**Figure 5:**
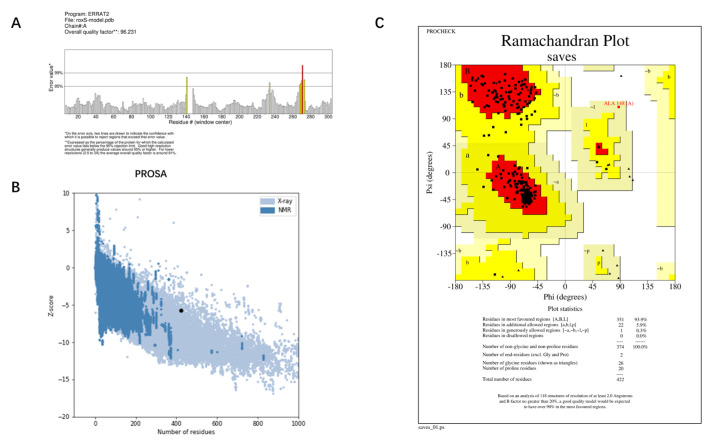
Quality evaluation results of Model provided by Alphafold: (**A**) Overall quality factor of ERRAT; (**B**) PROSA-web Z-scores of all protein chains; (**C**) PROCHECK analysis of the Ramachandran plot. The Overall quality factor of ERRAT > 95% means the structure has good resolution. The distribution of >90% of the residues in the most favorable region of the Ramachandran plot indicates that the protein structure is reasonable. The energy of the target protein structure is considered reasonable if the Z-score values of the target protein are distributed within the region of the graph represented by the Z-score values of these known proteins in PROSA.

The best model—Model 1—provided by I-TASSER, and the structure predicted by AlphaFold were evaluated for quality using ERRAT, PROCHECK, and PROSA. The Overall quality factors of Model 1 predicted by I-TASSER and the structure predicted by AlphaFold in ERRAT were 82.127% and 96.2312%, respectively. The structure predicted by AlphaFold had a high resolution. The number of residues in the most favored regions of Model 1 predicted by I-TASSER and the structure predicted by AlphaFold were 292 (76.2%) and 351 (93.9%), respectively. The structure predicted by AlphaFold was reasonable. The Z-score of Model 1 predicted by I-TASSER was −3.34 and was at the edge of the graph area plotted by Z-score values. The Z-score of the protein predicted by Alphafold was −5.76, which is within the graph area plotted by the Z-score values of known proteins, and its energy was reasonable. Combining the results of the three evaluation systems, ERRAT, PROCHECK, and PROSA (Figure 4 and Figure 5), Model 1 predicted by I-TASSER failed the quality evaluation. The structure of the RoxS protein predicted in AlphaFold was of good quality and was used for further molecular docking and molecular dynamics simulations.

### 2.2. Molecular Docking Studies of RoxS and Candidate Drugs

The RoxSR system of *P. aeruginosa* and the RegBA system of *Rhodobacter capsulatus* are homologous proteins with high sequence identity and functional similarity [27]. Swem LR [28] reported that coenzyme Q1 is a potent inhibitor of RegB that binds to the heptapeptide sequence GGXXNPF in RegB. The active site is generally evolutionarily conserved, and this conserved sequence has been found in the RoxS sequence of *P. aeruginosa*. The DeepSite of PlayMolecule software was used to predict the three possible docking pocket center axes for the RoxS protein (Table 1). The three computer-predicted docking pockets were docked with coenzyme Q1 using XP docking. The docking scores were −4.784, −5.562, and −3.026, with pocket 2 having the best score. In addition, the amino acid residues of the conserved sequence GGXXNPF were located directly in pocket 2. Therefore, the selection of the docking pocket requires further evaluation. 

**Figure 6:**
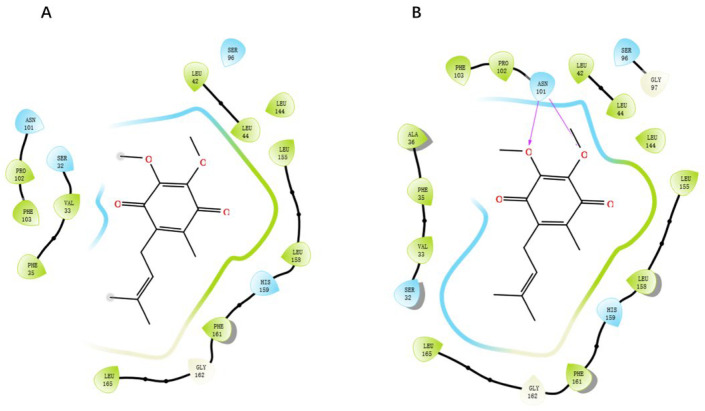
Two-dimensional interaction diagram of coenzyme Q1 and residues around pocket 2 (**A**), coenzyme Q1 and residues around pocket-GGXXNFP (**B**).

GGXXNPF residues on the RoxS protein were selected to generate a new docking pocket named pocket-GGXXNPF. The docking scores for pocket 2 and pocket-GGXXNPF were −5.562 and −6.089, respectively, using XP docking coenzyme Q1. Two-dimensional maps of protein–ligand interactions were observed, and in pocket-GGXXNPF, coenzyme Q1 formed hydrogen bonds with the key amino acid residue ASN101. In contrast, coenzyme Q1 entered pocket 2 and did not interact with the relevant amino acid residues. It simply fulfilled the principle of energetic complementation and did not fit into the binding structure or chemical environment (Figure 6). The docking score and amino acid residue interaction of pocket-GGXXNPF with coenzyme Q1 were consistent with those previously reported. Computer prediction results for pocket 2 also confirmed the superiority of pocket-GGXXNPF in terms of pocket volume and hydrophobicity in RoxS of *P. aeruginosa*. Therefore, by combining homology evidence from the literature and computational results, the conserved sequence GGXXNPF was selected as the center of this docking pocket. 

**Figure A1:**
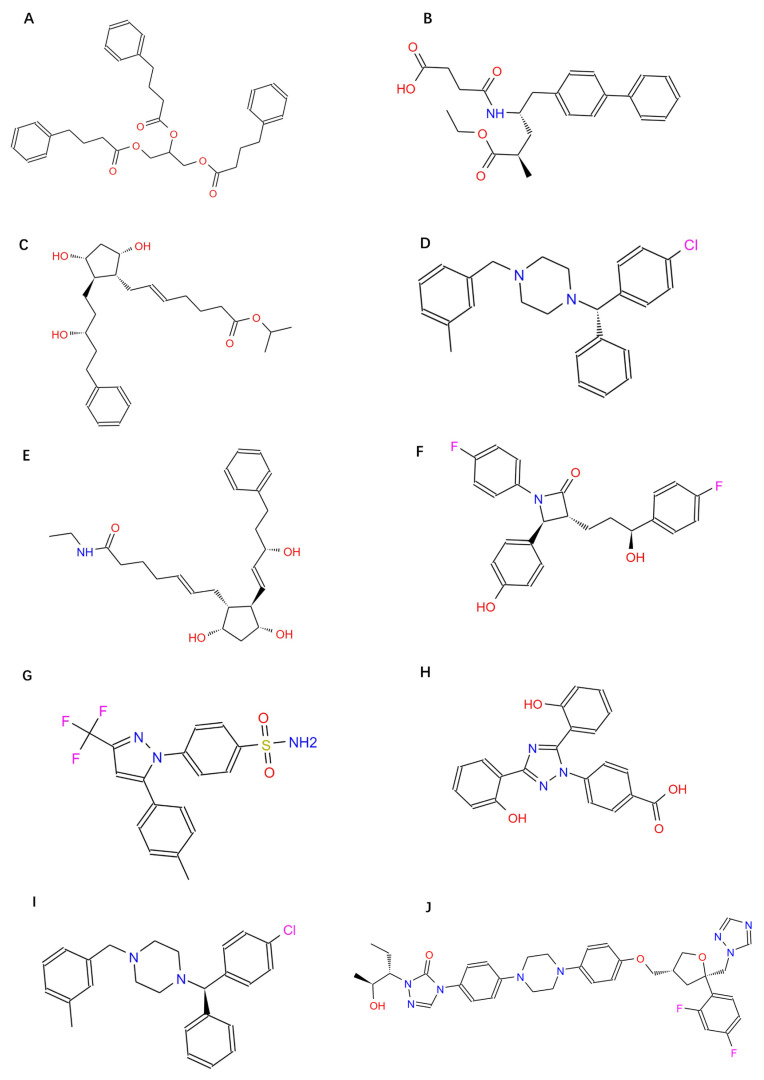
Structural formula of compounds with TOP 10 docking scores(**A**–**J**). The numbering of the compounds was consistent with that of Table 2.

**Figure A2:**
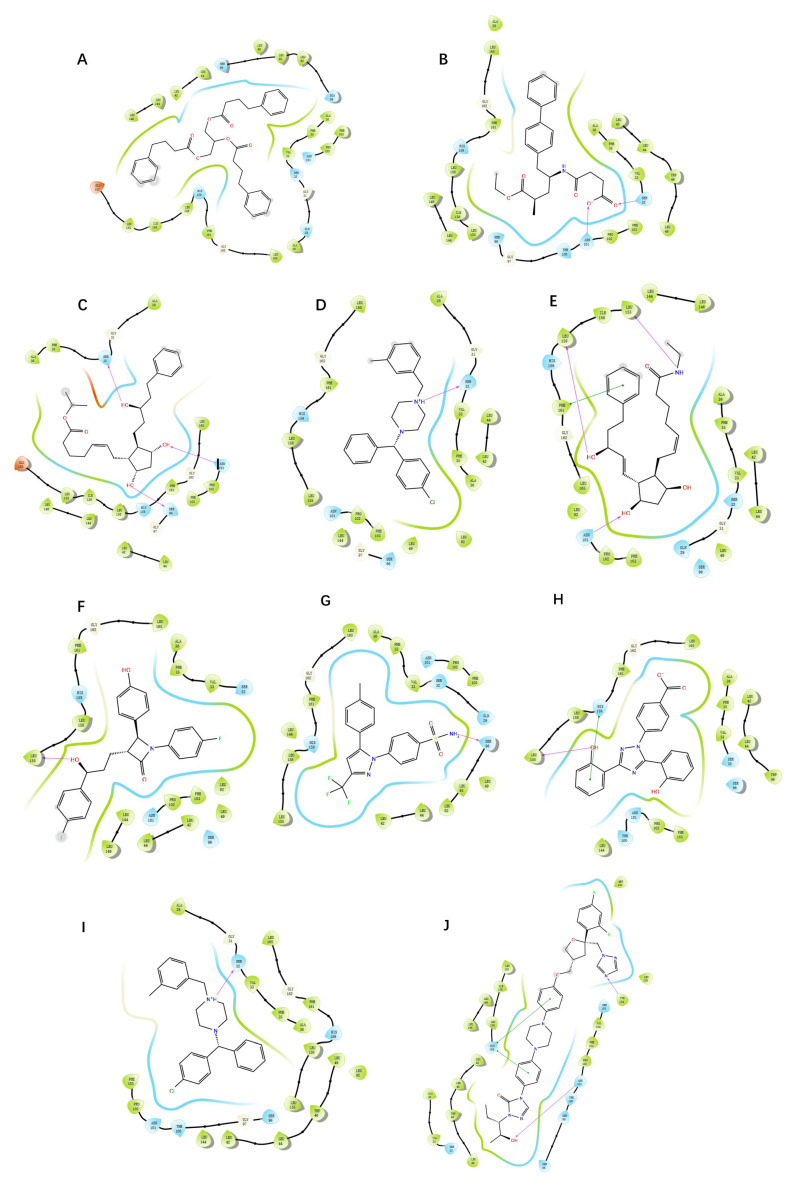
Two-dimensional interaction diagrams of compounds with TOP 10 docking scores (**A**–**J**). The purple arrows represent hydrogen bonding interactions and the green lines represent Pi-Pi stacking.

Molecular docking was performed on 1576 FDA-approved drugs to identify potential RoxS inhibitors. The higher the absolute value of a compound’s docking score, the better the compound binds to the active site of the target and may have better potential activity. After the “SP” docking was completed, the docking score was viewed and tallied. There were 152 drugs with docking scores less than −8, representing 9.7% of all docked drugs. These 152 drugs were docked again in XP docking mode, and the docking scores and docking poses were checked and counted. The 10 drugs with the highest scores in XP mode were screened (Table 2). The structural formulae and 2D interaction diagrams of the compounds are shown in Figure A1 and Figure A2 in Appendix B.

To select the most representative drugs, the 10 compounds obtained by docking were subjected to cluster analysis with the number of clusters being 3 (Table 3). Zetia (Ezetimibe, EB) and Noxafil (Posaconazole, PCZ) are representative drugs for Cluster 1 and Cluster 2, respectively. Celecoxib has been reported to be effective in combination with ampicillin against clinical isolates of *Pseudomonas aeruginosa* [29]. Therefore, Exjade (Deferasirox, DSX) was selected as the representative drug for Cluster 3. EB, DSX, and PCZ were chosen for further investigation. The best energy-docking conformations of these complexes were used as starting poses in molecular dynamics simulations.

### 2.3. Molecular Dynamics Simulations

**Figure 7:**
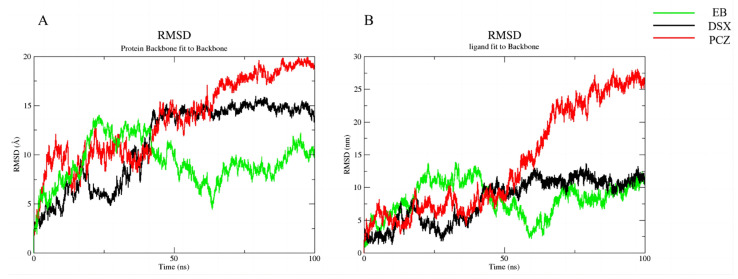
RMSD of protein backbone and ligand: (**A**) Protein Backbone fit to Backbone, (**B**) ligand fit to Backbone. EB, Ezetimibe; DSX, Deferasirox; PCZ, Posaconazole; RMSD, Root Mean Square Deviation.

**Figure 8:**
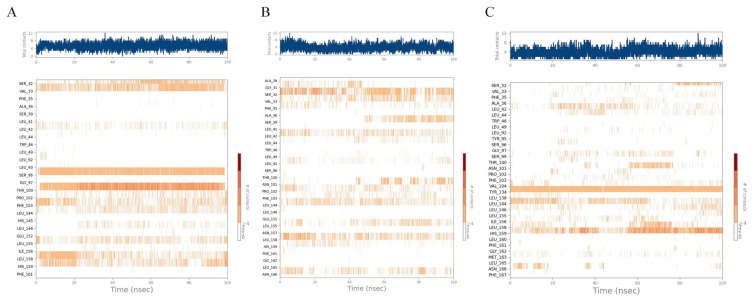
Statistical plot of ligand–protein interaction: (**A**) Ezetimibe; (**B**) Deferasirox; (**C**) Posaconazole.

To further explore the stability of target binding to candidate compounds, we performed molecular dynamics simulations. Molecular dynamics simulations of the complexes of all three candidate compounds with RoxS gradually reached equilibrium at approximately 75 ns. The fluctuation range of RMSD of the proteins and ligand was finally maintained within 3 Å, indicating that the binding of RoxS to the three candidate compounds we selected was stable (Figure 7 and Figure 8). 

First, EB formed very stable interactions with the VAL33, SER96, and THR100 amino acid residues, including hydrogen bonding and water-bridged interactions, which were present throughout the molecular dynamics simulation. The hydrogen bond formed by SER32 was also a key interaction. The formation of this hydrogen bond is a key factor in the formation of the stable conformation of EB. LEU144 and LEU146 also gradually formed hydrophobic interactions with EB after only 20 ns. Although these amino acid residues do not form stable interactions with EB, they play an important role in the transition from the starting to the final conformation of EB. DSX formed a stable interaction conformation faster than EB. The complex of DSX with RoxS reached stability around 40 ns, forming hydrogen bonds and hydrophobic interactions with SER32 and LEU144, respectively, which both existed stably throughout. After 40 ns, VAL33 and LEU155 gradually formed hydrophobic interactions with DSX, and SER39 and ASN101 formed water-bridging interactions with DSX. These interactions are key amino acid residues for the gradual formation of a stable conformation at later stages. PCZ formed a stable conformation with RoxS, hydrogen bonds with TYPR134, hydrophobic interactions with LEU144, water-bridged interactions, and hydrophobic interactions with HIS159. Molecular dynamics simulations revealed the gradual formation of water-bridged interactions with ASN101, LEU158, and finally a stable conformation.

In addition, the binding free energies of the three candidate compounds were calculated after the formation of stable conformations with RoxS. EB (−79.8150 ± 4.68 kcal/mol), DSX (−65.7558 ± −5.59 kcal/mol), and PCZ (−70.5598 ± −3.55 kcal/mol) all had satisfactory binding free energies, which are better than the binding free energy of the homologous protein inhibitor coenzyme Q1 (−56.0275 ± 2.83 kcal/mol) in complex with RoxS. The stability of the binding mode was further verified.

### 2.4. Individual Drug Candidates and Antibiotic Antibacterial Activity

The two drug-resistant strains of *P. aeruginosa* were sputum culture isolates obtained from the Laboratory Department of The Third Affiliated Hospital of Southern Medical University. According to drug sensitivity reports and the Latin American consensus, one strain was MDR *P. aeruginosa* and the other was XDR *P. aeruginosa* [30]. The MIC values of the three drug candidates and antibiotics were measured against the selected *P. aeruginosa* strains and the results are shown in Table 4. MDR *P. aeruginosa* was resistant to MEM, and XDR *P. aeruginosa* was severely resistant to AMK, according to the CLSI antibiotic threshold criteria [31]. None of the three drug candidates significantly inhibited either strain at any concentration tested.

### 2.5. Checkerboard Assays 

The activities of MEM and AMK in combination with EB, DSX, and PCZ were evaluated using checkerboard assays. The results are shown in Table 5. MEM was additive in combination with DSX or PCZ, respectively, and AMK and DSX were synergistic in combination.

**Figure 9:**
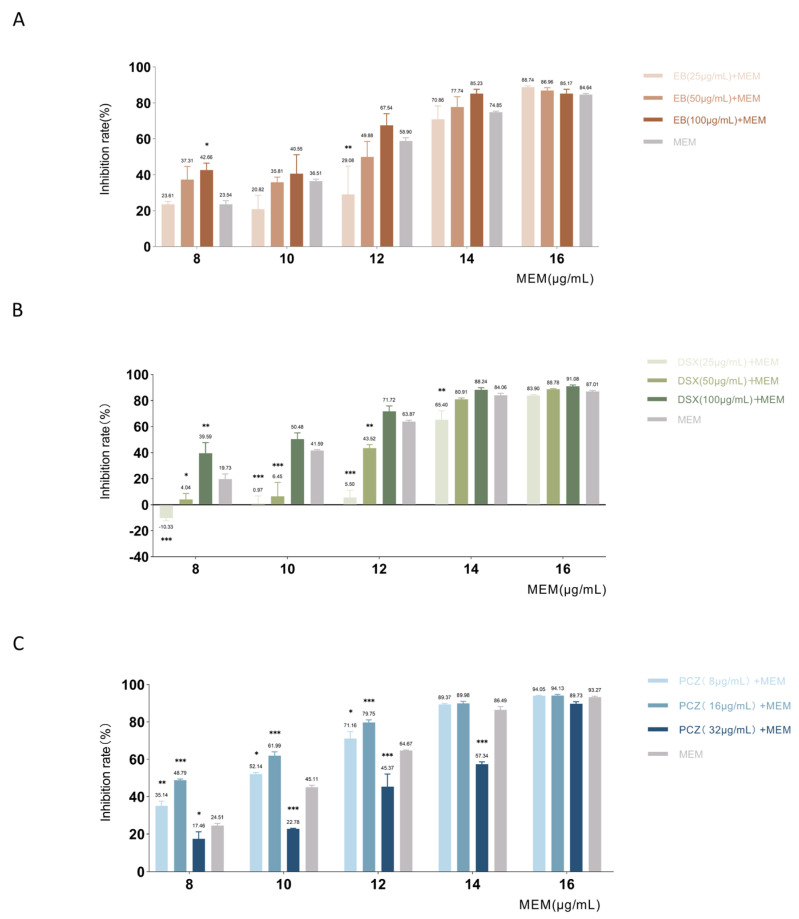
Combined antibacterial activity of Ezetimibe and meropenem (**A**), Deferasirox and meropenem (**B**), Posaconazole and meropenem (**C**). Legends: The vertical bar on each data point represents the standard error of the mean. The mean value of each group is located above the error bars. EB, Ezetimibe; DSX, Deferasirox; PCZ, Posaconazole; MEM, meropenem. * indicates *p*  ≤  0.05, ** *p*  ≤  0.01, *** *p*  ≤  0.001.

The results of MDR *P. aeruginosa* showed the combined effect of three drug candidates with MEM (Figure 9). In the checkerboard assays with EB, our results showed that 100 μg/mL of EB combined with 8 μg/mL of MEM had an enhancing effect on bacterial inhibition. The bacterial inhibition rate of 8 μg/mL MEM alone was 23.54%, and the combination of 100 μg/mL EB increased the inhibition rate to 42.66% *(p* = 0.03), which increased the inhibitory effect to 1.81 times that of MEM alone. In the checkerboard assays with DSX, MEM had an additive effect only in combination with 100 μg/mL DSX. The inhibition rate with 8 μg/mL of MEM alone was 19.73%, while the combined use of 100 μg/mL DSX increased the inhibition rate to 39.59% (*p* = 0.003), twice as effective as single-use. In a checkerboard test with PCZ, MEM in combination with 16 μg/mL PCZ showed the best inhibitory effect and reduced the MIC_50_. The combination of PCZ at 16 μg/mL increased the inhibition rate to 48.79% (*p* < 0.001), which was 1.99 times more effective than MEM alone. The trend was the same for higher concentrations of MEM in combination with PCZ.

**Figure 10:**
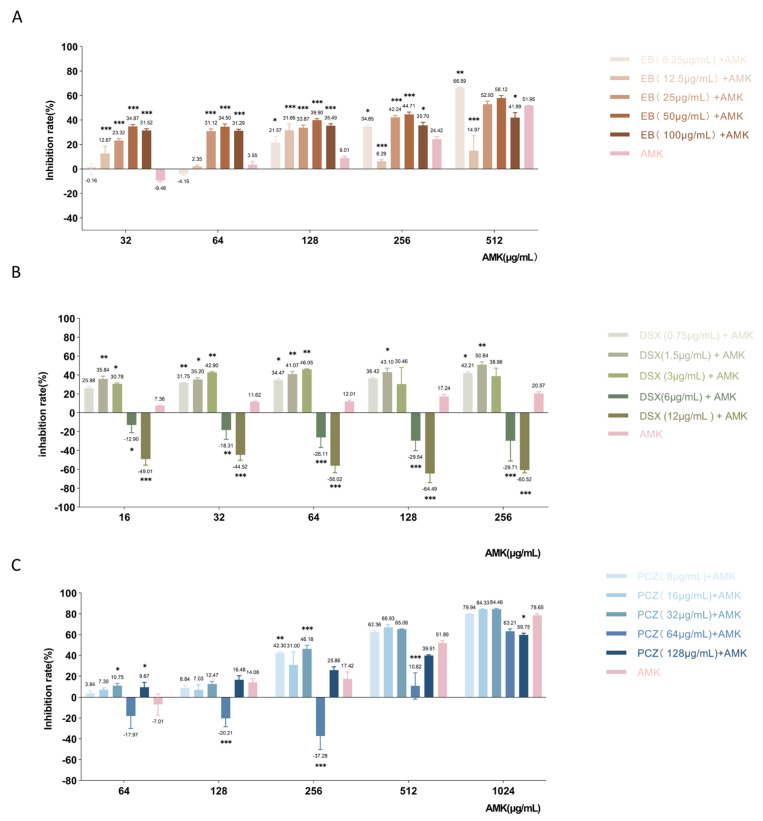
Combined antibacterial activity of Ezetimibe and Amikacin (**A**), Deferasirox and Amikacin (**B**), Posaconazole and Amikacin (**C**). Legends: The vertical bar on each data point represents the standard error of the mean. The mean value of each group is located above the error bars. EB, ezetimibe; DSX, Deferasirox; PCZ, Posaconazole; AMK, Amikacin. * indicates *p*  ≤  0.05, ** *p*  ≤  0.01, *** *p*  ≤  0.001.

To examine the combined inhibition of the three drug candidates with AMK, the checkerboard assays were performed in XDR *P. aeruginosa* (Figure 10). In the checkerboard assay with EB, the combination of 50 μg/mL EB had the best inhibitory effect when the concentration of AMK was 32 μg/mL to 256 μg/mL. The inhibition rate of 32 μg/mL AMK alone was −9.46% and had no inhibitory effect on XDR *P. aeruginosa*. This concentration of AMK in combination with 50 μg/mL EB increased the inhibition rate to 34.87% (*p* < 0.001). Approximately the same trend was seen in the results of 64 μg/mL and 128 μg/mL AMK in combination with EB; however, when the AMK concentration was 512 μg/mL, the effect was dramatically increased in combination with 6.25 μg/mL EB. In the checkerboard assays with DSX, our results showed that the combination of AMK with 0.75 μg/mL to 3 μg/mL of DSX significantly increased the inhibition rate, AMK combined with 1.5 μg/mL DSX has a synergistic effect; however, 6 μg/mL and 12 μg/mL of DSX in combination with AMK had an antagonistic effect. The bacterial inhibition rate of 16 μg/mL AMK alone was 7.36%, and the combination with 1.5 μg/mL DSX was optimal, increasing the inhibition rate to 35.84% (*p* = 0.006); this combination was 4.86 times more effective than single use. The effect of AMK at 64 μg/mL to 256 μg/mL combined with DSX showed the same trend as 16 μg/mL AMK. AMK at 32 μg/mL and 64 μg/mL in combination with DSX at 3 μg/mL had the best bacterial inhibitory effect. In the checkerboard assays of PCZ and AMK, the bacterial inhibition rate was −7.01% with 64 μg/mL AMK alone and 10.75% (*p* = 0.03) and 9.67% (*p* = 0.04) with the combination of 32 μg/mL and 128 μg/mL PCZ, respectively, however, the inhibitory effect was not significant. Only when 256 μg/mL of AMK was combined with PCZ the effect was seen. The inhibition rate of AMK alone was 17.42%, and the combination of PCZ with 32 μg/mL was the most effective, with the inhibition rate increased to 46.18% (*p* < 0.001), which was 2.65 times higher than that of the combination. Additionally, the combination of AMK with 64 μg/mL PCZ had an antagonistic effect.

In conclusion, all three compounds enhanced bacterial inhibition when combined with MEM and AMK. The combination of 100 μg/mL DSX and 16 μg/mL PCZ with MEM had additive effects, and the combination of 1.5 μg/mL DSX and AMK had synergistic effects, showing some potential for reversing resistance in *P. aeruginosa*. Although EB did not have additive or synergistic effects with the antibiotics, it increased the rate of bacterial inhibition. 

## 3. Discussion

*P. aeruginosa* presents a therapeutic challenge because of its inherently low susceptibility to a number of antibiotics and strong ability to develop antibiotic resistance [32]. Combination therapy is a sustainable option for reducing bacterial resistance [33]. Unfortunately, routinely used antibiotics are often ineffective against MDR and XDR strains, even with combination therapies [34]. Since TCS is currently found in bacteria but not in humans or other mammals, it can be used as a novel target for antibacterial drug action [35]. RoxS is a promising target for bacterial inhibition, however, little research has been conducted on its inhibitors. Virtual screening was performed for the target in this study to improve the efficiency of inhibitor screening. To better represent the impact in the real clinic, samples of strains from the clinic were selected to validate the in vitro activity of the drug candidates.

EB is a potent inhibitor of cholesterol absorption and has been approved for the treatment of hypercholesterolemia [36,37]. EB has potential antibacterial activity because it contains a beta-lactam ring [38]. Our results showed that in MDR *P. aeruginosa*, 8 μg/mL MEM combined with 100 μg/mL EB increased the inhibition rate by 1.81 times that of antibiotics alone. In XDR *P. aeruginosa*, the combination of EB inhibited bacterial growth, while AMK had no inhibitory effect. There have been no reports of EB being used to treat *P. aeruginosa* infections; however, the combination therapy of EB with MEM or AMK may be an alternative therapeutic strategy for multi-drug-resistant *P. aeruginosa* infections in patients with preexisting hyperlipidemia to reduce the dose of antibiotics and side effects, while lowering blood lipid levels. 

DSX is an FDA-approved iron chelator for the treatment of iron overload that has been used clinically for over 40 years [39]. *P. aeruginosa* requires high levels of iron during infection [40]. Several studies have shown that high iron concentrations facilitate biofilm formation and increase *P. aeruginosa* resistance to tobramycin and tigecycline [41]. Thus, DSX may be an effective treatment for lung diseases caused by biofilm-resistant *P. aeruginosa* [42]. This study confirmed that the combination of DSX and MEM had an additive effect, whereas the combination of DSX and AMK had a synergistic inhibitory effect. Combined with previous reports, this study further supports the contention that FDA-approved iron chelators can be developed for the treatment of multi-drug-resistant microbial infections. Meanwhile, DSX may also be a dual-target inhibitor that interferes with iron metabolism on one side and RoxS on the other.

Increased growth of fungal microbiota is a common side effect of antibiotic therapies. Prolonged use of antibiotics affects the growth of bacterial microbiota, which in turn causes secondary infections and leads to the proliferation of fungal microbiota [43]. The airways of patients with cystic fibrosis are susceptible to colonization by various microorganisms, particularly *Aspergillus fumigatus* and *P. aeruginosa* [44,45]. Mixed microbial infections usually occur in the lungs and can lead to airway inflammation and the worsening of lung function [46,47]. PCZ is useful in the treatment of Aspergillus-associated cystic fibrosis [48]. The results of this study show that PCZ in combination with MEM has an additive effect; therefore, the combination of PCZ and MEM has potential advantages in patients with multiple microbial infections in the lungs and displays resistance reversal in MDR *P. aeruginosa*.

The in vitro data showed that *P. aeruginosa* remained resistant to the drug combination treatment; however, the results of the present study are beneficial. The combination of 100 μg/mL DSX or 16 μg/mL PCZ with MEM had additive effects, whereas the combination of 1.5 μg/mL DSX and AMK had a synergistic effect. The combination therapy improved the inhibition of MDR and XDR *P. aeruginosa*. Even in cases where the antibiotics had no inhibitory effect, the combination showed some inhibition. This suggests that the selected combination strategy plays a role in reversing drug resistance in *P. aeruginosa*, and has great potential to fight infections in certain populations because any small or modest increase in antimicrobial activity with combination therapy may contribute to clinical success and recovery in actual clinical practice [49]. 

It is difficult to compare administered concentrations derived from in vitro studies with in vivo concentrations due to the influence of pharmacokinetics in humans; however, the results of the literature survey preliminarily indicate that the combination strategies screened in this study show some safety and clinical significance. The peak concentration in human plasma after injection of a standard dose of 1 g MEM is approximately 30 μg/mL [50]. When MDR or XDR *P. aeruginosa* causes pneumonia, AMK can be administered by nebulization to achieve high blood levels at the site of infection while avoiding the side effects of high-dose systemic administration [51]. At a dose of 400 mg, bid, AMK concentrations in the lungs can reach 976.1 μg/mL [52]. AMK is sufficient to treat meningitis at drug concentrations of 200 μg/mL in the central nervous system [53]. The concentrations of MEM and AMK used in this study were within the effective concentrations in vivo but did not exceed the toxic concentrations. The dosing safety of DSX and PCZ was assessed based on doses and cytotoxic concentrations in the literature because they are not antibacterial agents. Moreau-Marquis et al. reported that the recommended clinical dose of DSX is 20 mg/kg/day, which is equivalent to 280 μg/mL administered in vitro [54]. The dose of DSX used in this study was ≤100 μg/mL, which is lower than the daily dose used in clinical practice. No significant cytotoxicity was observed at PCZ concentrations below 100 μM (70.07 μg/mL) [55]. The results of in vitro experiments showed an additive effect of 16 μg/mL PCZ in combination with MEM, which was much lower than the toxic concentration.

The limitations of this study were that potential RoxS inhibitors were only initially screened by computational techniques, as well as the limited number of strains selected for the experiments. This target still has a lot of room for exploration. We will increase samples of clinical strains and structurally modify three FDA drugs as lead compounds to explore compounds with stronger inhibitory effects. Through molecular dynamics simulations, we found that SER32 and ASN101 were important for the formation of stable conformations of the compounds. Although no interaction between these two amino acids and the ligand was found in the docking results, these two amino acids guided the conversion of the ligand to a stable conformation, providing a key idea for further modification of the compound. In addition, RoxS is present not only in *P. aeruginosa* but also in other bacteria homologous to *P. aeruginosa*, including *Rhodobacter capsulatus*, *Syringae pseudomonas*, and so on. The combination strategy explored in this study may also have potential efficacy in other bacterial infections, which merits further investigation.

## 4. Materials and Methods

### 4.1. Homology Modeling

The RoxS protein sequences were retrieved from the NCBI database in the FASTA format. Swiss-Model (https://swissmodel.expasy.org/, accessed on 16 July 2022), I-TASSER (https://seq2fun.dcmb.med.umich.edu//I-TASSER/, accessed on 27 June 2022), and Alphafold (https://alphafold.ebi.ac.uk/, accessed on 27 June 2022) were utilized to design the three-dimensional structure of the proteins. The stereochemical quality, accuracy of the predicted models, and energy rationality were observed using PROCHECK (https://www.came.sbg.ac.at/prosa.php, accessed on 27 June 2022), ERRAT, and PROSA in SAVES (https://saves.mbi.ucla.edu/, accessed on 27 June 2022), respectively. Based on the evaluation scores, the best protein model with acceptable quality was selected.

### 4.2. Molecular Docking Studies of RoxS

#### 4.2.1. Identification of Docking Pockets

The predicted protein structure contained no ligand and could not generate a docking pocket from the self-containing ligand. In this study, a combination of computer predictions and homologous protein active sites was used to determine the docking pocket. The literature reported a conserved active site, GGXXNPF, in the evolution of RoxS, while further identifying the docking pocket in conjunction with the results of the prediction of the protein docking pocket in DeepSite in Playmolecule (https://www.playmolecule.com/deepsite/, accessed on 27 June 2022).

#### 4.2.2. Molecular Docking

The RoxS protein and 1576 FDA-approved drugs retrieved from the ZINC drug repository were prepared using Protein Prep Wizard [56] and LigPrep [57] in Maestro, respectively, followed by Ligand Docking [58]. Compounds with a docking score of less than eight were shortlisted using the standard precision mode (SP docking) and then re-docked using Extra Precision (XP) docking. More detailed and accurate information regarding the docking analysis was displayed using an XP Visualizer. Compounds with the TOP 10 docking scores were clustered into three groups based on volume overlap to screen out representative compounds, and the linkage method of calculation was weighted centroids.

### 4.3. Molecular Dynamics Simulation Analysis 

The molecular dynamics were determined using Desmond [59]. The dynamic system was first constructed using the SPC water model by dissolving the complex in a cube box filled with water adding appropriate amounts of Na+ ions and Cl− ions to neutralize the charge of the system and an additional 0.15 M concentration to simulate the real protein environment. We then embedded the complex in the POPE membrane model. Based on the OPLS4 force field [60], an energy minimization of 200 ps was first performed. The system was fully relaxed before the formal MD, and, finally, the MD calculation was performed at 300 K and 1 atm pressure for 100 ns. And the script thermal_mmgbsa.py script provided by Schrödinger was used to calculate the binding free energy values of the complexes in the steady state. Default settings are used except for the parameters mentioned.

### 4.4. MIC and Checkboard Assays

#### 4.4.1. MIC Assays

The antimicrobial efficacy of the drugs was determined using the broth microdilution method of the Clinical and Laboratory Standards Institute (CLSI). On a sterile ultra-clean bench, 100 μL of MH broth was added to each well of the 96-well plate, followed by 100μL of drugs (≤2% final concentration for DMSO and ≤4% final concentration for Tween 80 due to the low solubility of FDA drugs. The ratios of DMSO and Tween 80 are shown in the Table A2 in Appendix A) which were two-fold dilutions. At last, 100 μL standard bacterial suspension (5 × 10^5^ CFU/mL, 0.5 McFarland’s standard) was added. After incubation at 37 °C for 16–20 h, the OD_600 nm_ of the 96-well plates was measured in an enzyme marker. And the inhibition rate was calculated (see Appendix C for calculation formulas) to determine the minimum inhibitory concentration required to inhibit the growth of 50% of the bacterium (MIC_50_). Three rows of experimental wells, one row of the positive control group and one row of the negative control group, were placed in each 96-well plate. FDA drugs require additional pure drug groups to exclude absorbance effects owing to their low solubility. The concentrations of DMSO and Tween 80 in the medium of the control group were the same as those in the experimental group. Each experiment was repeated at least thrice.

#### 4.4.2. Checkerboard Assays for Antibiotic and Drug Candidates

In each 96-well plate, the drug candidate and antibiotic were diluted in a multiplicative series (<0.1% final concentration for DMSO and <0.2% final concentration for Tween 80) and combined in different concentrations to obtain 100 μL of mixed solution. Then, 100 μL of the bacterial suspension at a final concentration of 5 × 10^5^ CFU/mL was added, and the final volume of liquid per well was 200 μL. The concentrations of DMSO and Tween 80 in the medium of the control group were the same as those in the experimental group. Each checkerboard test was repeated at least three times. The plates were incubated, and the final OD600 was determined as described above. Differences between the combination and antibiotic-only groups (*p* < 0.05) were assessed using two-way analysis of variance (ANOVA), followed by Fisher’s least significant difference test for multiple comparisons.

The fractional inhibitory concentration index (FICi) was calculated as FIC of drug A (FICA) + FIC of drug B (FICB) [61], where FICA = MIC of drug A in combination/MIC of drug A alone, and FICB = MIC of drug B in combination/MIC of drug B alone. FICi was interpreted as follows: synergism = FICi ≤ 0.5; antagonist = FICi ≥ 4; additive = FICi > 0.5 and ≤1; indifference= 1 < FICi < 4. 

## 5. Conclusions

In this study, potential RoxS inhibitors were identified by virtually screening FDA-approved drugs. Our results suggested that Ezetimibe, Deferasirox, and Posaconazole are potential inhibitors of RoxS; in addition, the results of the in vitro experiments showed that combinations of these three drugs with meropenem or amikacin are promising antibacterial strategies that are expected to reverse *P. aeruginosa* resistance and provide new options for the treatment of clinically MDR and XDR *P. aeruginosa.*

## Figures and Tables

**Table 1:** Docking Pocket Centers for Deepsite Predictions.

Name	Scores	Centers
Pocket 1	0.998974845	[−29.90999984741211, −5.239999771118164, 23.329999923706055]
Pocket 2	0.998739839	[24.09000015258789, 0.7599999904632568, −38.66999816894531]
Pocket 3	0.988030121	[0.09000000357627869, 2.759999990463257, −8.670000076293945]

**Table 2:** Compounds with TOP 10 docking scores in XP mode.

Serial Number	ZIND ID	Docking Score
A	ZINC000038945666(Ravicti)	−11.249
B	ZINC000003792417(Sacubitril)	−10.294
C	ZINC000012468792(Xalatan)	−10.255
D	ZINC000019594557(Meclizine)	−10.148
E	ZINC000004474405(Latisse)	−10.084
F	ZINC000003810860(Zetia)	−9.988
G	ZINC000002570895(Celebrex)	−9.837
H	ZINC000001481815(Exjade)	−9.756
I	ZINC000019361042(Meclizine)	−9.602
J	ZINC000003938482(Noxafil)	−9.396

**Table 3:** Clustering results of compounds with TOP 10 docking scores.

Cluster	Serial Number	Drug Candidates
Cluster 1		
	F	ZINC000003810860(Zetia)
Cluster 2		
	J	ZINC000003938482(Noxafil)
Cluster 3		
	G	ZINC000002570895(Celebrex)
	H	ZINC000001481815(Exjade)
	B	ZINC000003792417(Sacubitril)
	C	ZINC000012468792(Xalatan)
	E	ZINC000004474405(Latisse)
	A	ZINC000038945666(Ravicti)
	D	ZINC000019594557(Meclizine)
	I	ZINC000019361042(Meclizine)

**Table 4:** MIC_50_ of drug candidates and antibiotics.

Drugs	MDR *P. aeruginosa*	XDR *P. aeruginosa*
	MIC_50(μg/mL)_	MIC_50(μg/mL)_
MEM	16	/
AMK	/	512
EB	>1600	>1600
DSX	>1600	>1600
PCZ	>512	512

Legends: MIC, minimum inhibitory concentration; MIC50, MIC that inhibited 50% of isolates; EB, Ezetimibe; DSX, Deferasirox; PCZ, Posaconazole; MEM, meropenem; AMK, Amikacin. MDR, multidrug-resistant; XDR, extensively drug-resistant. “/” is used to indicate that they were not tested.

**Table 5:** FICi Values of drug combinations.

Drug Combinations	FICi Value for *P. aeruginosa* Strain:	
	MDR *P. aeruginosa*	XDR *P. aeruginosa*
MEM + EB	>1.00	/
MEM + DSX	0.83–0.90	/
MEM + PCZ	0.83–0.86	/
AMK + EB	/	>1.00
AMK + DSX	/	0.50
AMK + PCZ	/	>1.00

Legends: Results are shown to 2 decimal places. FICi was and interpreted as follows: Synergism = FICi ≤ 0.5; antagonist = FICi ≥ 4; additive = FICi > 0.5 and ≤1; indifference = 1 < FICi < 4. EB, Ezetimibe; DSX, Deferasirox; PCZ, Posaconazole; MEM, meropenem; AMK, Amikacin. MDR, multidrug-resistant; XDR, extensively drug-resistant; FICi, the fractional inhibitory concentration index. “/” is used to indicate that they were not tested.

## Data Availability

Data presented in this study are available on request from the corresponding author. The data are not publicly available due to the samples in this study being secondary specimens from clinical patients.

## Appendix A

**Table A1:** Results predicted by Swiss-Model.

Model	GMQE	QMEAN	Oligo State	Template	Seq Identity
Model 1	0.28	0.54 ± 0.05	Homo-dimer	4biv.1.A	22.12%
Model 2	0.28	0.55 ± 0.05	Homo-dimer	5idj.1.A	22.75%
Model 3	0.28	0.54 ± 0.05	Homo-dimer	4biu.1.A	22.12%
Model 4	0.28	0.55 ± 0.05	Homo-dimer	4biv.1.A	22.12%
Model 5	0.27	0.54 ± 0.05	Homo-dimer	4e02.1.B	19.63%
Model 6	0.26	0.54 ± 0.05	Homo-dimer	6paj.1.A	21.63%

Legend: GMQE, Global Model Quality Estimate; QMEAN, Qualitative Model Energy Analysis.

**Table A2:** Each drug solution in MIC assays was prepared.

FDA-Approved Drugs	The Ratio of DMSO and Tween in the Highest Concentration Drug Solution
Ezetimibe	2% final concentration for DMSO and 4% final concentration for Tween 80
Deferasirox	2% final concentration for DMSO and 4% final concentration for Tween 80
Posaconazole	2% final concentration for DMSO and 1% final concentration for Tween 80

Legend: FDA, Food and Drug Administration; DMSO, Dimethyl sulfoxide; MIC, minimum inhibitory concentration.

## Appendix C

Formula (A1). The formula for calculating the inhibition rate of the three FDA-approved drugs was as follows:

(A1) $$inhibition\;rate\left(\%\right)=\frac{\left[{OD}_{positive\;group}-\left({OD}_{experimental\;group}-{OD}_{pure\;drug\;groups}\;\right)-{OD}_{negative\;group}\right]}{{OD}_{positive\;group}-{OD}_{negative\;group}}\times 100\%$$

Formula (A2). The inhibition rate of antibiotics was calculated as follows:

(A2) $$inhibition\;rate\left(\%\right)=\frac{{OD}_{positive\;group}-{OD}_{\;experimental\;group}}{{OD}_{positive\;group}-{OD}_{negative\;group}}\times 100\%$$

Note: OD, optical density.
